# Uremic serum-induced calcification of human aortic smooth muscle cells is a regulated process involving Klotho and RUNX2

**DOI:** 10.1042/BSR20190599

**Published:** 2019-10-11

**Authors:** Ashish Patidar, Dhruv K. Singh, Shori Thakur, Ken Farrington, Anwar R. Baydoun

**Affiliations:** 1 School of Life and Medical Sciences, University of Hertfordshire, College Lane, Hatfield AL10 9AB, Hertfordshire, U.K.; 2 Renal Unit, Lister Hospital, Coreys Mill Lane, Stevenage SG1 4AB, U.K.; 3 Faculty of Health and Life Sciences, De Montfort University, The Gateway, Leicester LE1 9BH, U.K.

**Keywords:** Chronic kidney disease, Human aortic smooth muscle Cells, Klotho, RUNX2, Uraemic serum, Vascular calcification

## Abstract

Vascular calcification (VC) is common in subjects with chronic kidney disease (CKD) and is associated with increased cardiovascular risk. It is an active process involving transdifferentiation of arterial smooth muscle cells (SMCs) into osteogenic phenotype. We investigated the ability of serum from CKD subjects to induce calcification in human SMCs *in vitro* (calcific potential of sera: CP), and associated changes in expression of Runt-related transcription factor 2 (RUNX2), SM22α, and Klotho. Sera from subjects with CKD (18 stage 3, 17 stage 4/5, and 29 stage 5D) and 20 controls were added to human cultured SMCs and CP quantified. The CP of CKD sera was greater (*P<0.01*) than that of controls, though not influenced by CKD stage. Modification of diet in renal disease estimated glomerular filtration rate (MDRD-4 eGFR) (*P<0.001*), serum phosphate (*P=0.042*), receptor activator of nuclear factor κappa-B ligand (RANKL) (*P=0.001*), parathyroid hormone (PTH) (*P=0.014*), and high-density lipoprotein (HDL)/cholesterol ratio (*P=0.026*) were independent predictors of CP accounting for 45% of variation. Adding calcification buffer (CB: calcium chloride [7 mM] and β-glycerophosphate [7 mM]) increased the CP of control sera to approximate that of CKD sera. CP of CKD sera was unchanged. CKD sera increased RUNX2 expression (*P<0.01*) in human SMCs and decreased SM22α expression (*P<0.05*). Co-incubating control but not CKD serum with CB further increased RUNX2 expression (*P<0.01*). Both SM22α and Klotho expression decreased significantly (*P<0.01*) in the presence of CKD serum, and were virtually abolished with stage 5D sera. These findings support active regulation by CKD serum of *in vitro* VC by induction of RUNX2 and suppression of SM22α and Klotho.

## Introduction

Vascular calcification (VC) in chronic kidney disease (CKD) may contribute to the high prevalence of cardiovascular disease burden in this patient group by promoting decreased vascular compliance, left ventricular hypertrophy and arterial narrowing and obstruction [1]. The formation of VC in the CKD setting involves the differentiation of smooth muscle cells (SMCs) to assume a bone-like phenotype and eventual deposition of hydroxyapatite crystals in the vicinity of extracellular elastin and/or collagen. This is an active process involving osteo/chondrogenic de-differentiation of SMCs when exposed to high calcium and/or phosphate *in vitro* [2].

The precise cellular pathways that lead to VC in CKD are not fully elucidated. Inducers of oxidative stress including hydrogen peroxide, reactive nitrogen and/or oxygen species [3], homocysteine [4], C-reactive protein (CRP) [5], uremic toxins such as *p*-cresyl sulphate and indoxyl sulphate [6], and chronic glucosative stress, can result in the SMCs losing their phenotypic characteristics and gaining a secretory osteo/chondrogenic phenotype. The transformed cells release molecules which may accelerate the progression of VC, and their presence in serum may confer the potential to induce VC. We have termed this phenomenon as calcific potential (CP) of serum and demonstrated that serum from subjects with advancing CKD induced a graded calcification on rat aortic SMCs cultured *in vitro* [7].

We have also demonstrated, using the same rat SMC model, that sera from subjects with diabetes has the potential to induce calcification [8]. This suggests that both CKD and diabetes milieu confers CP to serum, enabling it to induce calcification even outside the disease setting. All these findings however relate to rat SMCs. It is not clear whether the same CP is evident in human cells which would be more clinically relevant as VC does show some species differences especially pertaining to potential biomarkers which may mediate this process. Osteoprotegerin (OPG), for instance, has been implicated as protecting against VC in mice [9] and rats [10], while potentially promoting calcification in humans with high levels expressed adjacent to calcified atherosclerotic lesions [11] and in haemodialysis subjects with *in vivo* VC [12]. These findings suggest that the process and mediators of VC may be different in rodents and in humans. This raises the question of whether the CP of serum demonstrated previously in rat SMCs [7,8] may be evident in human SMCs. Also, we have limited information on the mechanism(s) of serum-induced CP, which we have now proceeded to study.

In the present paper, we have investigated whether serum from CKD patients induces calcification of human aortic SMCs and the relationship of its CP with the severity of CKD. Additionally, we have examined whether CKD sera regulates the expression of SM22α, a marker of SMC plasticity, Runt-related transcription factor 2 (RUNX2), an osteoblastic transcription factor, and of Klotho, an anti-ageing protein which protects against VC. The data from the current study along with our previous observations [7,8] have enabled us to identify not just important species differences but also identification of biomarker profiles with the potential to predict individuals at risk of VC. These novel findings could have important clinical relevance in providing opportunities for identification and therefore early clinical interventions in CKD subjects at risk of calcification.

## Materials and methods

### Patient recruitment

Patients were recruited from the renal clinics in East and North Herts NHS Trust. Inclusion and exclusion criteria were as described previously [7] and patients were classified into three groups based on eGFR:

- Moderate CKD (eGFR 30–60 ml/min/1.73 m^2^ – CKD stage 3 (CKD3); 18 subjects)
- Advanced CKD (eGFR <30 ml/min/1.73 m^2^ but not on dialysis – CKD stages 4 and 5 (CKD4/5); 17 subjects)
- Haemodialysis (CKD stage 5 dialysis (HD); 29 subjects who had undergone at least 3 months of dialysis). These patients were assumed to have a GFR of 0.

Twenty age- and sex-matched healthy individuals with normal renal function, without diabetes, CKD, or major illnesses were recruited as controls.

### Data collection and routine biochemical analysis

Data collection and biochemical analysis of samples together with analysis of specific biomarkers were carried out as reported previously [7].

### Culture of human aortic SMCs

Human SMCs were purchased from Cell Application (Cat: 354-05a) and cultured under standard conditions in Dulbecco’s Modified Eagle Medium (DMEM) supplemented with 10% (v/v) foetal bovine serum (FBS), 100 Unit.ml^−1^ penicillin and 100 μg.ml^−1^ streptomycin (complete culture medium). Confluent monolayers were routinely passaged using 0.01% trypsin and either subcultured or used for experimentation.

### Induction of calcification of human SMCs

A total of 60–70% confluent monolayers of cells between passages 3 and 8 were incubated for 1–7 days with calcification buffer (CB) consisting of 7 mM CaCl_2_ and β-glycerophosphate (β-GP) in the complete culture medium to determine the optimum calcification period. The CP of human serum samples from control and CKD settings was also determined by incubating cells with 10% serum in complete culture medium. In parallel studies, CB was added together with 10% serum to establish whether the CP of the latter could be enhanced within a calcifying milieu. Sera at 10% (v/v) concentration was diluted in DMEM that was buffered with 37 g/l of bicarbonate and in 5% CO_2_ in an *in-vitro* cell culture incubator, thus maintaining the pH at physiological levels. Calcification was determined using the DICA-500 Ca^2+^ assay kit as described previously [7].

### Western blotting

To determine whether calcification of human SMCs was associated with changes in expression of Klotho or RUNX2, cell lysates were generated from monolayers incubated with 10% serum for 5 days and subjected to Western blotting as described by Baydoun et al. (1999) [13] using selective anti-Klotho (1:1000 dilution) or anti-RUNX2 (1:1000 dilution) antibodies. Lysates were also analysed using an anti-SM22α antibody (1:1000 dilution) to determine whether the SMCs differentiated following calcification. Protein bands were semi-quantified by densitometry using ImageJ software.

### Materials

Cell culture reagents and other general biochemical reagents including CaCl_2_ and β-GP were purchased from Sigma–Aldrich (London, U.K.). Penicillin and streptomycin were from Fisher Scientific (Loughborough, U.K.), and FBS from Gibco (Scotland, U.K.). The bicinchoninic acid assay (BCA) protein quantification kit was from Thermo Scientific (Basingstoke, U.K.) and the DICA-500 Ca^2+^ assay kit was from Universal Biologicals (Cambridge, U.K.). Anti-Klotho, anti-RUNX2, anti-SM22α, and horseradish peroxidase (HRP)–conjugated secondary antibody (anti-rabbit) were from Abcam (Cambridge, MA). Anti-biotin was from Cell Signalling (Boston, MA) and HRP–conjugated anti-β actin was from Sigma (St. Louis, MO).

### Statistical analysis

Statistical Package for Social Sciences (SPSS) version 21 (SPSS Inc., Chicago, U.S.A.) was used for data analysis. For continuous variables, the significance of differences between two groups was determined by independent *t* test (for normally distributed data) and the Mann–Whitney *U*-tests or Wilcoxon test (for non-normally distributed data). One-way ANOVA was computed with subsequent Dunnett’s post hoc testing and the Kruskal–Wallis test were employed to explore the significance of differences in multiple groups as appropriate. Categorical data were tested using χ^2^ test. Correlations were computed using Pearson’s or Spearman’s tests as appropriate. For multivariate analysis, values of CP or biochemical variables were log-transformed for skewed distributions.

## Results

### Demographics and clinical factors

All subjects were Caucasian and did not show significant differences in age or gender ratios across study groups. A high proportion of patients (54%) with CKD had diabetes. The proportion was similar across the three CKD groups. Systolic and diastolic blood pressure (BP) were significantly less in the HD compared with the moderate and advanced CKD cohort or controls (Table 1).

**Table 1:** Biochemical parameters of serum from controls and CKD subjects

VARIABLES	GROUPS				STATISTICS		
	Control (C)	Moderate CKD3 (M)	Advanced CKD4/5 (A)	Haemodialysis (HD)	M v A	M v HD	A v HD
					*P*-value		
**Number**	20	18	17	29	-	-	-
**Age (years)**	63.3 ± 6.2	62.0 ± 8.4	66.7 ± 17.7	63.7 ± 14.8	NS	NS	NS
**Gender (M/F)**	12/8	13/5	9/8	20/9	-	-	-
**SBP (mmHg)**	149 ± 24	145 ± 13	153 ± 28	134 ± 17*	NS	0.025	<0.01
**DBP (mmHg)**	87 ± 9	85 ± 10	89 ± 12	73 ± 11^‡^	NS	=0.001	<0.05
**Creatinine (μmol/l)**	72 ± 17	149 ± 30	291 ± 83^‡^	735 ± 223^‡^	<0.001	<0.001	<0.001
**eGFR (MDRD-4)**	94 ± 24	43 ± 8^‡^	20 ± 5^‡^	0^‡^	<0.001	<0.001	<0.001
**Urea (mmol/l)**	5.3 ± 1.2	10.2 ± 2.3^†^	20.4 ± 6.2^‡^	20.5 ± 5.7^‡^	<0.001	<0.001	NS
**Haemoglobin (g/dl)**	14.0 ± 1.0	12.5 ± 2.6*	11.2 ± 2.1^‡^	11.0 ± 1.1^‡^	NS	<0.01	NS
**High CRP%**	11	21	14	54			
**Calcium (mmol/l)**	2.48 ± 0.13	2.40 ± 0.12	2.36 ± 0.10*	2.37 ± 0.20*	NS	NS	NS
**Cholesterol (mmol/l)**	5.8 ± 1.0	4.8 ± 1.1*	4.6 ± 1.0^†^	4.1 ± 1.2^‡^	NS	NS	NS
**HDL/cholesterol (mmol/l)**	0.26 ± 0.05	0.25 ± 0.05	0.31 ± 0.1	0.31 ± 0.1	<0.05	<0.05	NS
**Phosphate (mmol/l)**	1.01 ± 0.15	1.15 ± 0.21	1.31 ± 0.23^†^	1.64 ± 0.47^‡^	<0.05	<0.001	=0.01
**Albumin (g/l)**	45.3 ± 2.5	40.9 ± 4.8^‡^	41.5 ± 4.7^†^	37.2 ± 2.8^‡^	NS	<0.01	<0.001
**BAP (IU/l)**	-	25.6 ± 6.6	28.7 ± 7.0	23.6 ± 6.3	NS	<0.001	<0.05
**PTH (pmol/l)**	4 (2, 6)	3 (1, 23)	6 (2, 18)	18 (1, 44)	NS	<0.001	<0.001
**OPG (pmol/l)**	4.0 ± 1.5	5.5 ± 1.9	6.1 ± 1.9*	9.9 ± 3.8^‡^	<0.001	<0.001	<0.001
**MGP (ng/ml)**	15.4 ± 1.4	-	-	1.90 ± 0.7	-	-	-

Differences between controls and CKD groups are denoted by symbols. Differences between CKD groups appear in three columns on the left-hand side of the Table. Abbreviations: BAP, bone-specific alkaline phosphatase; DBP, diastolic BP; eGFR (ml/min/1.73 m^2^), estimated glomerular filtration rate calculated using MDRD-4 formula; HDL, high-density lipoprotein; MGP, matrix gla protein; NS, non-significant; RANKL, receptor activator of nuclear factor κ-B ligand; SBP, systolic BP. *P<0.05. ^†^*P<0.01*. ^‡^*P<0.001* denote significant difference from controls by ANOVA using post-hoc Dunnett’s test.

### Baseline biochemistry and haematology

Table 1 shows predictable differences in modification of diet in renal disease estimated glomerular filtration rate (eGFR MDRD-4) and in CRP between the cohorts in that the former declined and the latter increased with advancing CKD. Relative to controls, serum levels of urea, creatinine, phosphate, and OPG also increased with advancing kidney disease, while albumin levels reduced most markedly in the HD cohort. PTH levels also increased, most evident in the CKD4/5 and HD cohorts. Haemoglobin levels were significantly lower in all CKD groups than controls and lower in the HD compared with the CKD3 but not the CKD4/5 group. Serum calcium and cholesterol were also lower in all CKD groups compared with controls but did not differ between CKD groups. The high-density lipoprotein (HDL)/cholesterol ratio was however higher in CKD4/5 and HD than in controls and CKD3. Bone alkaline phosphatase (BAP) levels were lower in HD than in other CKD groups. Matrix gla protein (MGP) also showed lower expression in the HD group but data for the other CKD groups were not available. These trends are similar to those described previously using the same serum samples [7].

### *In vitro* model of calcification of human SMCs

**Figure 1:**
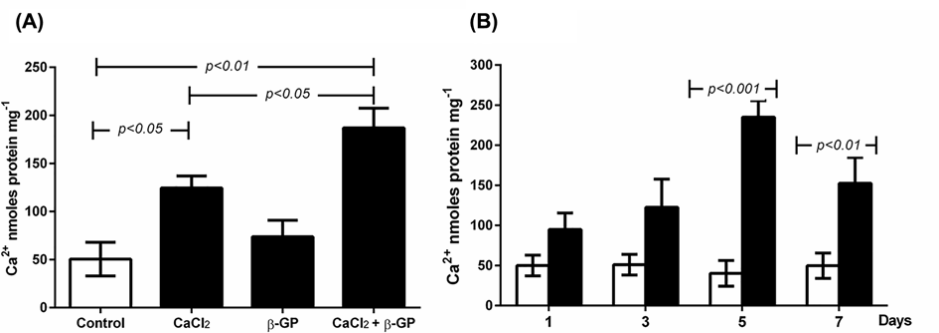
Induction and time course of calcification in human SMCs Human SMCs cultured to ∼60% confluence were incubated in complete culture medium alone (Control; open bar), media containing 7 mM CaCl_2_, 7 mM β-GP (solid bars) or a combination of both (**A**). The time course of calcification using CaCl_2_ and β-GP combined is shown in (**B**). Calcification was quantified and normalised for total cell protein as described in the ‘Materials and methods’ section. The data are presented as means ± S.E.M. of three independent experiments with five replicates in each.

Consistent with trends seen in the rat SMCs [7], incubation of human SMCs with 7 mM CaCl_2_ in complete culture medium for 5 days resulted in ∼two-fold more calcification compared with control (*P<0.05*). Addition of β-GP on its own at the same concentration did not induce significant calcification. The combination of CaCl_2_ and β-GP at 7 mM resulted in approximately three- to four-fold more calcification than control (*P<0.01*). This was significantly higher (*P<0.05*) than that seen with CaCl_2_ alone (Figure 1A). This effect was maximum on day 5, declining by day 7 (Figure 1B). These observations confirmed that human SMCs respond to calcification inducers and express a pattern of calcification similar to that seen in rat SMCs [7].

### Effects of uremic serum on calcification of human SMCs

**Figure 2:**
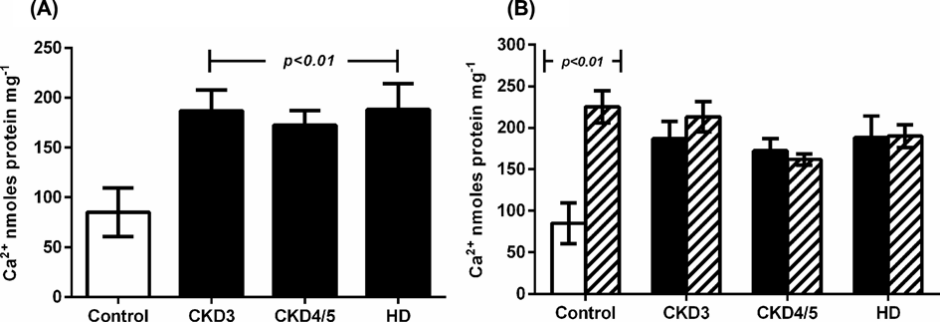
Effects of serum on calcification of human SMCs Human SMCs cultured to ∼60% confluence were incubated for 5 days in complete culture medium with 10% serum from controls (open bars, (**A**)) or from CKD3, CKD4/5, or HD cohorts (solid bars, (A)). In parallel, cells were also incubated with serum and CB together (hatched bars, (**B**)). Calcification was quantified and normalised for total cell protein as described in the ‘Materials and methods’ section. The data are presented as means ± S.E.M. of three independent experiments with five replicates in each.

Consistent with data obtained using rat aortic SMCs, the CP of CKD sera in human SMCs was greater than the CP of control sera (*P<0.001*) (Figure 2A). In contrast, the responses in the human SMCs, unlike those seen in rat SMCs, were not graded relative to CKD severity, and there were no statistical differences in the CP of sera from CKD3, CKD4/5, and HD. Furthermore, addition of CB to serum only increased calcification in control cells (*P<0.001*) and not in cells treated with sera from any of the CKD cohorts (Figure 2B). These results contrast with our initial findings in rat aortic SMCs [7], reflecting potential species differences.

### Relationships of serum calcification potential to demographic, clinical, and biochemical variables

*In vitro* serum CP correlated positively with serum creatinine, blood urea, OPG, RANKL, and phosphate but negatively with eGFR, haemoglobin, serum albumin, and MGP (Table 2). The relationship with age, gender, diabetes, systolic BP (SBP), diastolic BP (DBP), HDL/cholesterol ratio, BAP, and PTH were not significant in univariate analysis. However, the best multivariate model included eGFR (*P<0.001*), serum phosphate (*P=0.042*), HDL/cholesterol ratio (*P=0.026*), PTH (*P=0.014*), and RANKL (*P=0.001*) as independent predictors of serum CP. The model predicted 45% of the variance (Table 3).

**Table 2:** Correlation coefficients of *in vitro* CP of uremic serum in human SMCs with biomarkers

VARIABLES	COEFFICIENT (*P-value*)
**eGFR (MDRD-4)**	−0.574 (*P<0.001)*
**Serum creatinine**	0.382 (*P<0.001)*
**Blood urea**	0.507 (*P<0.001)*
**Serum phosphate**	0.381 (*P<0.001)*
**Haemoglobin**	−0.388 (*P<0.001)*
**Serum albumin**	−0.369 (*P<0.001)*
**Serum OPG**	0.309 (*P<0.010)*
Serum RANKL*	0.442 (*P<0.010*)
Serum MGP^*,†^	-0.538 (*P<0.001)*

Calcification potential values were correlated with particular biochemical variables using Pearson’s or Spearman(*) correlation as appropriate. Estimated glomerular filtration rate (eGFR: ml/min per 1.73 m^2^) was calculated using MDRD-4 formula. ^†^Correlation was only performed in control and HD cohort.

**Table 3:** Multivariate predictors of serum calcification potential

Adjusted R^2^ = 0.450	Unstandardized coefficients		Standardized coefficients	*t*	*P*-value
	B	Std. error	β		
**(Constant)**	171.324	34.408		4.979	0.000
**eGFR (ml/min/1.73 m^2^)**	0.908	0.197	−0.513	−4.603	0.000
**Serum phosphate (mmol/l)**	36.320	17.531	0.220	2.072	0.042
**HDL/total cholesterol ratio**	−139.928	61.718	−0.0196	−2.267	0.026
**Serum PTH (pmol/l)**	−1.491	0.591	−0.252	−2.523	0.014
**Serum RANKL (pmol/l)**	174.939	51.371	0.302	3.405	0.001

### Expression of Klotho, RUNX2, and SM22α expression in human SMCs

**Figure 3:**
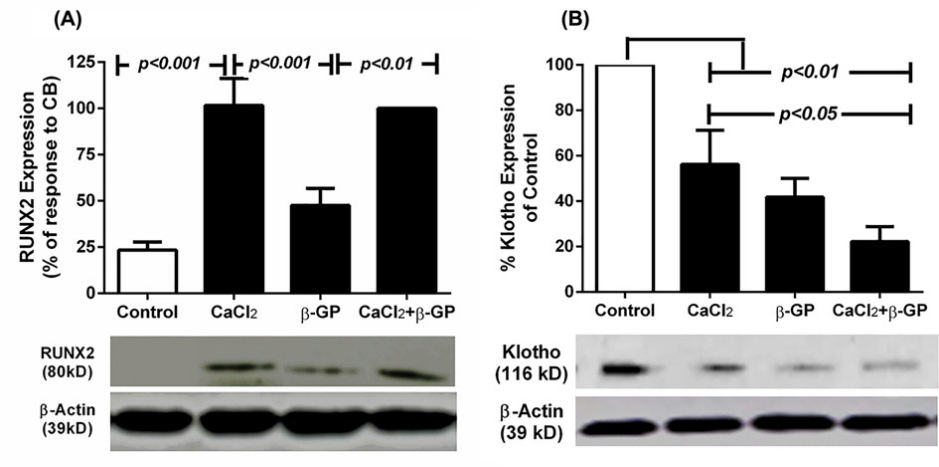
Expression of RUNX2 and Klotho in human SMCs in the presence of calcification inducers Human SMCs cultured to ∼60% confluence were incubated in complete culture medium alone (Control; open bar), media containing 7 mM CaCl_2_, 7 mM β-GP, or a combination of both (solid bars). Cell lysates were collected and probed for RUNX2 (**A**) or Klotho (**B**) using Western blotting as described in the ‘Materials and methods’ section. The data are presented as means ± S.E.M. of at least three independent experiments and expressed as percentages relative to maximal expression in cells incubated with CB for RUNX2 or control for Klotho expression.

To establish whether expression of Klotho, RUNX2, and SM22α altered following the different treatment conditions, we initially determined the expression profiles of each protein in control cells. The Western blots generated show that expression of RUNX2 in SMCs was minimal under control conditions. Levels were significantly enhanced following 5 days incubation with CaCl_2_ (*P*<0.001; Figure 3A) but only marginally with β-GP. Incubations with CB for the same period enhanced RUNX2 levels well above controls (*P*<0.001) and β-GP treatment (*P*<0.01), but similar to those with CaCl_2_ alone (Figure 3A). In contrast, calcification induced by CB was higher than that of CaCl_2_ alone (Figure 1) suggesting that factors additional to RUNX2 may contribute to the greater CP of CB. Klotho expression was found to be constitutive and significantly inhibited by CaCl_2_, β-GP, and CB, which respectively reduced Klotho protein levels to ∼50, 40, and 20% of control levels (*P<0.01*; Figure 3B).

**Figure 4:**
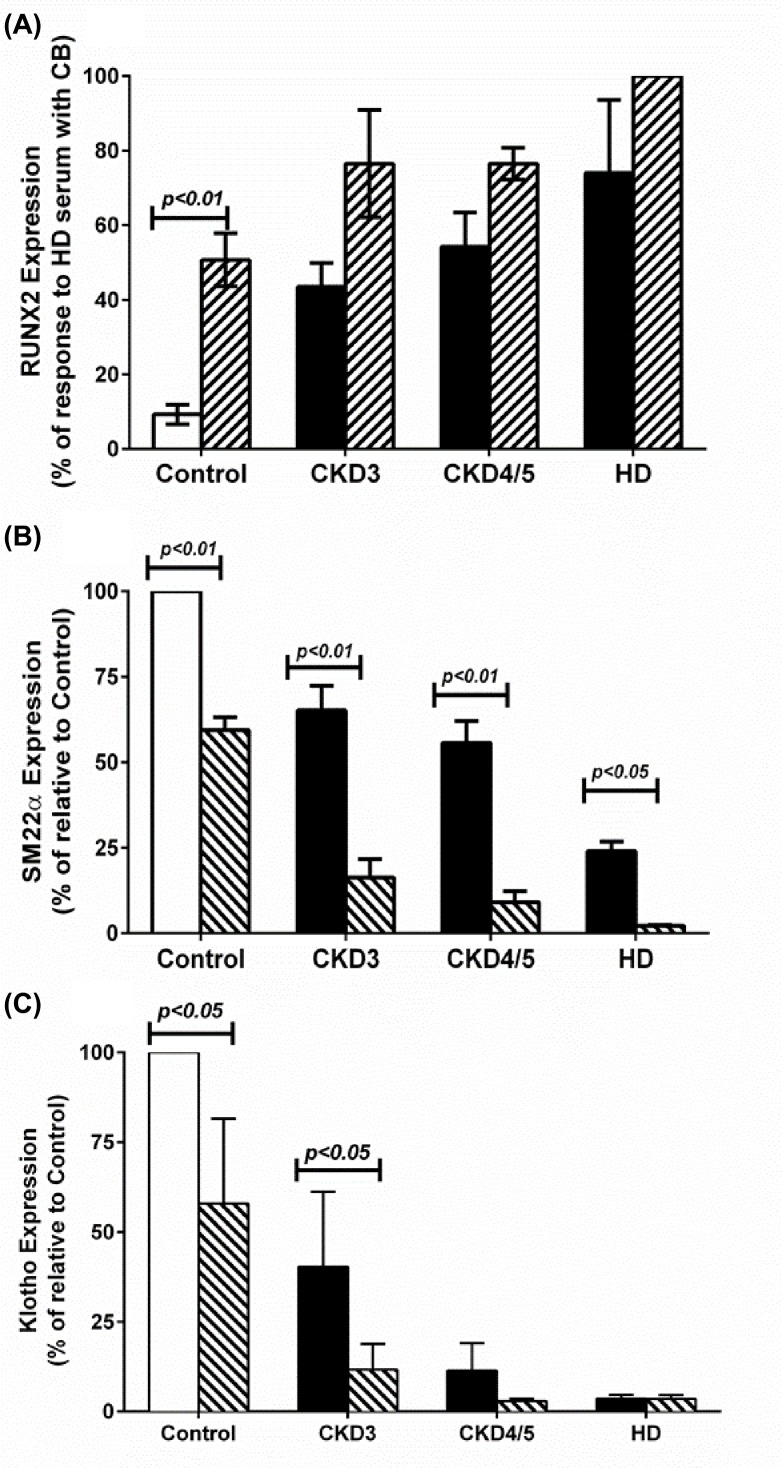
Effects of serum on the expression of RUNX2, SM22α, and Klotho in human SMCs Human SMCs cultured to ∼60% confluence were incubated for 5 days in complete culture medium with serum in the absence (open and black bars) and presence of CB (7 mM CaCl_2_ plus 7 mM β-GP) (hatched bars). Cell lysates were collected and probed for RUNX2 (**A**), SM22α (**B**), or Klotho (**C**) using Western blotting as described in the ‘Materials and methods’ section. The data are presented as means ± S.E.M. of at least three independent experiments and expressed in percentages relative to maximal expression in cells incubated with HD serum for RUNX2 or control serum for SM22α and Klotho expression.

Pooled serum from controls induced minimal RUNX2 expression while pooled serum from CKD3, CKD4/5, and HD caused induction of RUNX2 expression which was significantly higher than controls (*P<0.01* in all cases). Increments in RUNX2 expression tended to reflect the degree of kidney failure, though the differences between CKD groups were not significant (Figure 4A). Upon supplementation of serum with CB, RUNX2 expression increased across all groups but was only statistically significant in cells incubated with control serum (*P<0.01*; Figure 4A).

In parallel studies, SM22α expression decreased significantly (*P<0.05*) and in a graded manner when incubated with uremic serum, decreased to ∼70% of control expression with CKD3, ∼65% with CKD4/5, and ∼25% with HD sera. Addition of CB together with serum samples caused further reduction in SM22α expression, decreasing levels to ∼55% with control serum (*P<0.01*), to less than 25% with CKD3 (*P<0.01*) and CKD4/5 (*P<0.01*) sera, and to almost complete suppression with HD serum (Figure 4B).

With regards to Klotho, expression was significantly inhibited by serum from CKD3, CKD4/5, and HD subjects when compared with control serum (*P<0.01* in all cases; Figure 4C). There was a decreasing trend in Klotho expression with increasing renal failure in that serum from HD subjects inhibited Klotho expression more significantly compared with CKD3 (*P<0.05*). The difference between CKD4/5 and HD was not significant since inhibition of expression was near complete for both. The addition of CB caused further decrements in Klotho expression with control and CKD3 sera (Figure 4C).

## Discussion

The studies described above have focussed on establishing whether serum from CKD settings is able to cause calcification of human aortic SMCs similar to the CP observed in rat SMCs reported previously [7]. We studied aortic SMCs since calcification at arterial sites has been shown to impact on mortality and morbidity. We acknowledge though that calcification is widespread and occurs in other cell types of vascular and tissue origin. We have also investigated phenotypic changes in human SMCs caused by uremic serum and further determined whether expression of RUNX2 and/or Klotho are altered in order to gain insight into how serum might regulate VC in human SMCs.

A potential limitation of our study is the high proportion of the CKD cohorts which had diabetes. This should be borne in mind when interpreting our results since the presence of diabetes is also a potent stimulus to VC. However, our finding that there was no difference in calcification potential between diabetic and non-diabetic sera is reassuring.

Similar to previous findings in rat SMCs [7], is the observation that calcification of human SMCs followed the same time course of induction as rat SMCs. The addition of β-GP to human SMCs did not induce significant calcification while CaCl_2_ did. The combination further enhanced calcification, which was similarly maximal as seen in rat SMCs at 5 days incubation. Interestingly, incubation of human SMCs with CaCl_2_ or β-GP alone induced marginally higher calcification than in rat SMCs. By comparison, on addition of similar concentrations of CB, calcification in human SMCs was ∼30% less than rat SMCs [7]. There are some possible explanations, including species differences in serum calcium and phosphate levels. Physiological calcium levels are lower in humans than in the rat (2.2–2.5 vs 2.7–3.2 mmol/l). Phosphate levels are also lower in humans at 0.8–1.4 vs 1.8–2.2 mmol/l [14,15]. Hence under physiological conditions, rat SMCs may be adapted to be more resilient to the induction of calcification than human cells. This may infer the presence of protective factors. Increasing calcium and phosphate levels further by the addition of CB may be enough to overwhelm these protective mechanisms, leading to higher calcification.

Similar to the previous study in rat SMCs, sera from subjects with CKD showed *in vitro* CP in human SMCs which followed the same time course of induction as rat SMCs. However, the effect of sera on human cells did not differ with the severity of renal failure as rat SMCs. Maximal calcification was induced even with serum from the early-stage CKD. This may reflect a higher propensity of human SMCs to calcify compared with rat SMCs, further suggesting the presence of protective mechanisms in the rat.

Co-incubation with control sera and CB induced a similar degree of calcification to uremic serum. There was no increment in calcification in any of the CKD groups. These findings highlight other differences between human and rat SMCs. In the latter, co-incubation of CB with control serum caused no significant increase in CP, while CP was significantly increased with CKD3 and CKD4/5 sera to the level observed in HD in the absence of CB [7]. This further supports the notion that rat SMCs may have greater defence mechanisms under control conditions which, may be overridden by increasing calcium and phosphate availability. Other possibilities include the inability of some pro-/anti-calcific elements of human serum to act on rat SMCs due to species differences. These potential mechanisms warrant further investigation in future studies.

Correlations of serum *in vitro* CP with biochemical parameters in both rat and human SMCs suggest cross-species similarities. In multivariate modelling, serum levels of phosphate, RANKL, PTH, HDL/cholesterol ratio, and eGFR (MDRD-4) were significant predictors of *in vitro* CP and explained 45% of its variance. Interestingly, OPG levels predicted serum *in vitro CP* in rat but not in human SMCs while RANKL levels predicted *in vitro* CP in human SMCs but not in rat. Interestingly higher PTH levels emerge as protective against VC in our model. Mid-range PTH levels may well be protective since they induce a normal level of bone turnover in the setting of CKD where there is skeletal resistance to the action of PTH. High bone turnover induced by severe hyperparathyroidism liberates excess calcium from bones which may induce tissue calcification. Low levels of bone turnover (adynamic bone disease) prevent the bones acting as a sump for excess dietary calcium, including that associated with use of calcium containing phosphate binders. This excess calcium is then available for tissue calcification. Hence, depending on the management strategies for bone and mineral disease, ‘higher’ levels of PTH may well emerge as protective in models of calcification.

It has recently been shown that OPG [16], similar to vitamin-D [17], protected against calcification at lower levels while at higher supra-physiological levels contributed towards the progression of VC. This supports the finding that genetic deletion of OPG leads to VC [9]. However, most patients with diabetes [18] and/or CKD [19] may already have high levels of OPG. We found that serum OPG levels correlated positively with *in vitro* CP in CKD cohort. Hence, it is probable that OPG plays a diverse role in VC depending on the underlying pathology and its ambient levels. We did not measure the degree of *in vivo* calcification in CKD cohort, but it is reasonable to assume that HD subjects have higher VC compared with pre-dialysis patients as has been previously shown [20].

To confirm whether calcification in our experimental condition involved phenotypic osteo-/chondro-genic differentiation of human SMCs, we examined changes in RUNX2 and SM22α expression. Expression of Klotho was also investigated due to its critical role in VC. CaCl_2_ either alone or in combination with β-GP induced significant RUNX2 elevation while β-GP induced only marginally higher RUNX2 expression than controls. This contrasts with at least one report claiming that longer incubation with β-GP at 10 mM resulted in significant RUNX2 elevation [21]. The marginal effect of β-GP in the current studies may therefore, be due to the lower concentration (7 mM) and shorter duration of incubation. The observations with CaCl_2_ however, suggest that calcium is more potent compared with β-GP in inducing calcification and dedifferentiation. Interestingly, serum calcium levels change only minimally in CKD/HD compared with phosphate. This does not preclude a role for calcium in the induction of calcification as it is sequestered in cellular stores and may be released upon stimulation without any obvious change in serum calcium levels.

We also explored the regulation of Klotho expression under similar conditions to those described above. We found that calcification inducers inhibited Klotho expression. This suggests that the degree of calcification correlates with the extent of inhibition of Klotho expression, implying a protective role of Klotho in calcification. This is consistent with previous reports claiming that overexpression of Klotho provided protection against calcification [22]. The mechanisms of this Klotho mediated protection remain to be identified in our *in vitro* model. High levels of calcium and phosphate, in the presence of FBS, were able to further inhibit Klotho expression by ∼56 and ∼42%, respectively. CB further blunted Klotho expression to ∼22%. This suggests that both calcium and phosphate inhibit Klotho expression and that this inhibition is more pronounced when cells are incubated with CB.

We also found that Klotho expression was down-regulated with advancing renal disease, with sera from HD subjects virtually abolished its expression. This finding suggests the presence of factors other than calcium and phosphate in HD sera which can suppress Klotho and potentially enhance calcification. In this regard, previous research has shown that oxidative stressors and cellular senescence inhibit Klotho expression [23], providing a novel mechanism of serum-induced calcification. Oxidative stressors and cellular senescence can not only reduce Klotho expression but can also increase the phosphorylation of RUNX2 and actively recruit more calcium and phosphate intracellularly [3]. We therefore propose that Klotho down-regulation may be additionally regulated by oxidative stress. However, further research is required to confirm this hypothesis. Also, inhibition of Klotho expression may lead to systemic FGF-23 resistance which may impact on progression of calcification [22]. Due to limited serum availability we were not able to quantify FGF-23 in our cohort.

In parallel experiments, RUNX2 expression was up-regulated, and SM22α was down-regulated in the presence of uremic serum. The loss in SM22α suggests that uremic serum has the potential to transform contractile SMCs into secretory phenotype. This de-differentiation is mediated through myocardin, which regulates expression of SMC-specific genes [24,25]. It has been suggested that Klotho deficiency also leads to increased phosphorylation of RUNX2 [26] and that Klotho regulates the expression of Pit-1 and Pit-2 which are major transporters of phosphate in the cells. Hence, loss of Klotho can lead to an increase in calcification by increasing intracellular transport of phosphate and RUNX2 phosphorylation [27]. The critical role of Klotho in the protection of SMCs phenotypicity could be of considerable importance in understanding the pathobiology of VC.

## Conclusions

These studies have confirmed that serum from patients with CKD can induce calcification in human SMCs. The studies have also highlighted subtle species differences between human and rat SMCs in terms of the propensity to calcify under our study conditions. In addition, we have demonstrated for the first time that the effects of CKD sera are mediated at least in part via regulation of Klotho and RunX2 expression. Moreover, we have further validated our experimental system as a suitable *in vitro* model for screening serum CP which could be exploited in predicting risks of calcification *in vivo*. Further work is necessary to relate *in vitro* CP to *in vivo* VC at different stages of CKD – both cross-sectionally and over time.

## Perspectives

- **Background as to why the study was undertaken**: Studies were carried out to establish whether serum from subjects with CKD has potential to induce calcification of human aortic SMCs outside the disease milieu and identify cellular mediators of this process.
- **Brief summary of the results**: Sera from subjects with CKD possess CP and are capable of inducing calcification of human aortic SMCs *in vitro* through up-regulation of RUNX2 and suppression of Klotho expression.
- **The potential significance of the results to human health and disease**: The *in vitro* CP of CKD sera may be exploited to identify individuals at risk of VC, to facilitate definition of biomarker profiles, to predict calcification risk and to inform the development of potential novel therapeutic strategies for the management of patients at risk.

## Abbreviations

BAP: bone alkaline phosphatase
BP: blood pressure
CB: calcification buffer
CKD: chronic kidney disease
CP: calcific potential
CRP: C-reactive protein
FBS: foetal bovine serum
HDL: high-density lipoprotein
HRP: horseradish peroxidase
MDRD-4 eGFR: modification of diet in renal disease estimated glomerular filtration rate
MGP: matrix Gla protein
OPG: osteoprotegerin
PTH: parathyroid hormone
RANKL: receptor activat of nuclear factor κ-B ligand
RUNX2: Runt-related transcription factor 2
SMC: smooth muscle cell
VC: vascular calcification
β-GP: β-glycerophosphate
